# Enteric glial cells counteract *Clostridium difficile* Toxin B through a NADPH oxidase/ROS/JNK/caspase-3 axis, without involving mitochondrial pathways

**DOI:** 10.1038/srep45569

**Published:** 2017-03-28

**Authors:** Lara Macchioni, Magdalena Davidescu, Katia Fettucciari, Maya Petricciuolo, Leonardo Gatticchi, Davide Gioè, Vincenzo Villanacci, Massimo Bellini, Pierfrancesco Marconi, Rita Roberti, Gabrio Bassotti, Lanfranco Corazzi

**Affiliations:** 1 Department of Experimental Medicine, University of Perugia, Perugia, Italy; 2 Scientific and educational center of Terni, University of Perugia, Perugia, Italy; 3 Pathology Section, Spedali Civili di Brescia, Brescia, Italy; 4 Department of Gastroenterology, University of Pisa, Pisa, Italy; 5 Department of Medicine, University of Perugia, Perugia, Italy

## Abstract

Enteric glial cells (EGCs) are components of the intestinal epithelial barrier essential for regulating the enteric nervous system. *Clostridium difficile* is the most common cause of antibiotic-associated colitis, toxin B (TcdB) being the major virulence factor, due to its ability to breach the intestinal epithelial barrier and to act on other cell types. Here we investigated TcdB effects on EGCs and the activated molecular mechanisms. Already at 2 hours, TcdB triggered ROS formation originating from NADPH-oxidase, as demonstrated by their reduction in the presence of the NADPH-oxidase inhibitor ML171. Although EGCs mitochondria support almost completely the cellular ATP need, TcdB exerted weak effects on EGCs in terms of ATP and mitochondrial functionality, mitochondrial ROS production occurring as a late event. ROS activated the JNK signalling and overexpression of the proapoptotic Bim not followed by cytochrome c or AIF release to activate the downstream apoptotic cascade. EGCs underwent DNA fragmentation through activation of the ROS/JNK/caspase-3 axis, evidenced by the ability of ML171, N-acetylcysteine, and the JNK inhibitor SP600125 to inhibit caspase-3 or to contrast apoptosis. Therefore, TcdB aggressiveness towards EGCs is mainly restricted to the cytosolic compartment, which represents a peculiar feature, since TcdB primarily influences mitochondria in other cellular types.

The enteric nervous system (ENS) is a complex neural structure devoted to the control of uptake of nutrients, secretion, blood flow, motility, inflammatory, and immunological processes of the alimentary tract^1^. The ENS is mainly represented by two cell populations, neurons and enteric glial cells (EGCs), the latter being present in a ratio of approximately 4:1 compared to neurons^2^. The old simplistic concept of EGCs as support cells for the ganglia and/or nutritive elements for the neurons has radically changed in the last years. In fact, this cell population plays a pivotal role in the economy of the gut and, in addition to the mechanical support function^3^, exhibits trophic functions toward enteric neurons^4^,^5^, is involved in enteric neurotransmission^6^, has immunological functions^7^,^8^, and is involved in dysmotility^9^,^10^ and inflammatory^11^,^12^ conditions of the gut.

In view of their specialized functions, EGCs require an extensive energy demand that is covered by the mitochondrial oxidative phosphorylation. It is conceivable that pathogens alter mitochondrial functions, with the opportunistic aim to affect the fate of the infected host cell. Indeed, *Clostridium difficile* toxins, as well as other bacterial toxins, induce severe mitochondrial dysfunction in several cell types^13^,^14^,^15^,^16^. Therefore, the possible protective role of EGCs during enteric bacterial insult is attracting increasing interest.

*C. difficile* is a normal inhabitant of the gut microbiota of about 1–3% of adults. However, after pretreatment with a broad spectrum of antibiotics, *C. difficile* can take advantage of the lack of commensal bacteria and colonize the large intestine. Here, it produces the enterotoxins TcdA and TcdB, which elicit an inflammatory immune response that allows them to cross the mucosal membrane. One of the most direct events attributed to TcdA and TcdB is the ability to breach the intestinal epithelial barrier and act on other cell types. In this context, the protective role of EGCs is essential. We found previously that TcdB exherts cytopathic and cytotoxic effect on EGCs, producing cell cycle arrest and apoptosis, and increases cell sensitivity to inflammatory cytokines^17^. To extend this study, we investigated the molecular mechanisms activated by EGCs as defense strategy in response to TcdB. As an early event, the toxin triggered cytosolic ROS production and a downstream pathway culminating in DNA fragmentation of a EGCs pool. This mechanism did not involve mitochondrial factors, nor deeply altered mitochondrial functionality, but was restricted to the cytosolic compartment, involving a pathway that flows through a NADPH oxidase/ROS/JNK/caspase-3 axis.

## Results

### TcdB induced apoptosis in EGCs is mediated by ROS

**Figure 1:**
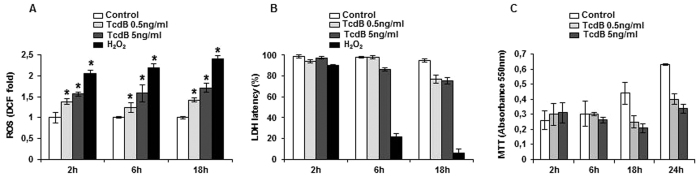
TcdB toxicity in EGCs. Cells were treated with of 0.5 or 5 ng/ml TcdB for the indicated times. (**A**) Fluorimetric quantitation of total ROS by staining with 10 μM CM-H_2_DCFDA. H_2_O_2_ (2 mM), positive control. (**B**) LDH latency. H_2_O_2_ (2 mM), positive control. (**C**) Cell viability, determined by MTT. Data are the mean ± SD of three independent experiments (*p < 0.01 versus control).

**Figure 2:**
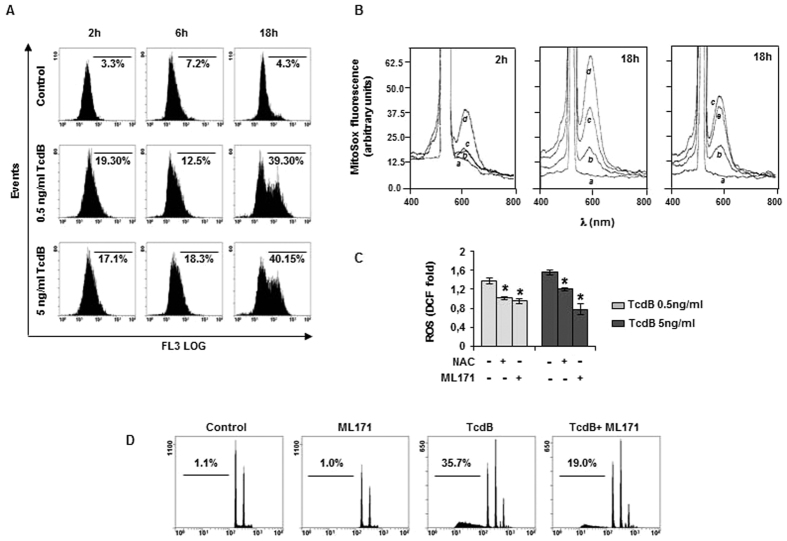
ROS involvement in TcdB induced apoptosis. EGCs were treated with 0.5 or 5 ng/ml TcdB for the indicated times. (**A**) Flow cytometry analysis of celluar ROS, determined as superoxide ion using 5 μM DHE. (**B**) Detection of mitochondrial ROS by MitoSox Red probe (5 μM). MitoSox fluorescence spectra (λ_ex_ 510 nm) in EGCs treated with 5 ng/ml TcdB: (**a**) probe; (**b**) control; (**c**) 5 ng/ml TcdB; (**d**) 60 μM antimycin, positive control; *e*, 5 ng/ml TcdB plus 10 μM ML171. (**C**) ML171 (10 μM) and NAC (1 mM) contrast ROS production induced by 2-hour treatment with TcdB. Total ROS were determined fluorimetrically by staining with 10 μM CM-H_2_DCFDA. Data are the mean ± SD of three independent experiments (*p < 0.01 versus control). (**D**) Effect of the NOX inhibitor ML171 on EGCs apoptosis induced by 18-hour treatment with 5 ng/ml TcdB. In A, B, and D, representative experiments out of three are shown. ML171 was supplied as DMSO solution to give 0.1% final concentration of the vehicle. We verified that this concentration does not influence any of the parameters measured.

Several cell types contrast pathogens by activating a respiratory burst. Since we observed previously that TcdB induced apoptosis in EGCs^17^, and confirmed here apoptosis already at 18-hour treatment (Supplementary Fig. S1), we investigated whether ROS act as an upstream signal in this process. Intracellular ROS levels were determined in EGCs treated with TcdB for 2–18 hours, by labeling cells with dichlorofluorescein (CM-H_2_DCFDA). A significant increase of total ROS was evidenced already at 2 hours, and persisted up to 18-hour treatment (Fig. 1A). Despite the early ROS production, no significant LDH leakage occurred at 2 and 6 hours TcdB treatment and more than 75% LDH latency was preserved at 18-hour treatment, indicating that the majority of cells maintain plasma membrane integrity (Fig. 1B). MTT reduction assays showed an increase of metabolic activity over time in control EGCs, whereas the activity remained almost constant in TcdB treated cells (Fig. 1C), probably as the balance between cell proliferation arrest and cell damage. Next, we investigated the contribution of the mitochondrial compartment to ROS production by using MitoSox Red and dihydroethidium (DHE) probes. Flow cytometric analysis showed an increase of FL3 channel fluorescence in response to DHE oxidation in treated cells, indicating the early production of total cellular superoxide ion that accumulated within 18 hours (Fig. 2A. Fluorimetric analysis of EGCs labeled with MitoSox Red showed that, compared to control, mitochondria-derived superoxide were unchanged after 2-hour treatment, but increased at 18 hours (Fig. 2B), left and intermediate panels, trace *c*). Since this increase was not significantly detected by the fluoresceine probe (Fig. 1A), it can be deduced that mitochondrial superoxide represents a small fraction of total ROS. The possible involvement of the NADPH oxidase (NOX) complex in TcdB-mediated ROS production^18^ was investigated by pretreatment of EGCs with the specific NOX inhibitor ML171, followed by 2-hour treatment with TcdB. Total ROS production was reduced by ML171. At the same time, the antioxidant N-acetylcysteine (NAC) was also effective in scavenging ROS (Fig. 2C). Preincubation of EGCs with ML171 did not prevent mitochondrial ROS production (Fig. 2B, right panel, traces *c* and *e*). We further examined if ROS elevation was involved in TcdB-induced apoptosis. The preincubation with ML171 resulted in a significant decrease in apoptotic cell death, demonstrating the role of NOX complex in EGCs apoptosis (Fig. 2D).

### TcdB treatment and mitochondrial impairment

**Figure 3:**
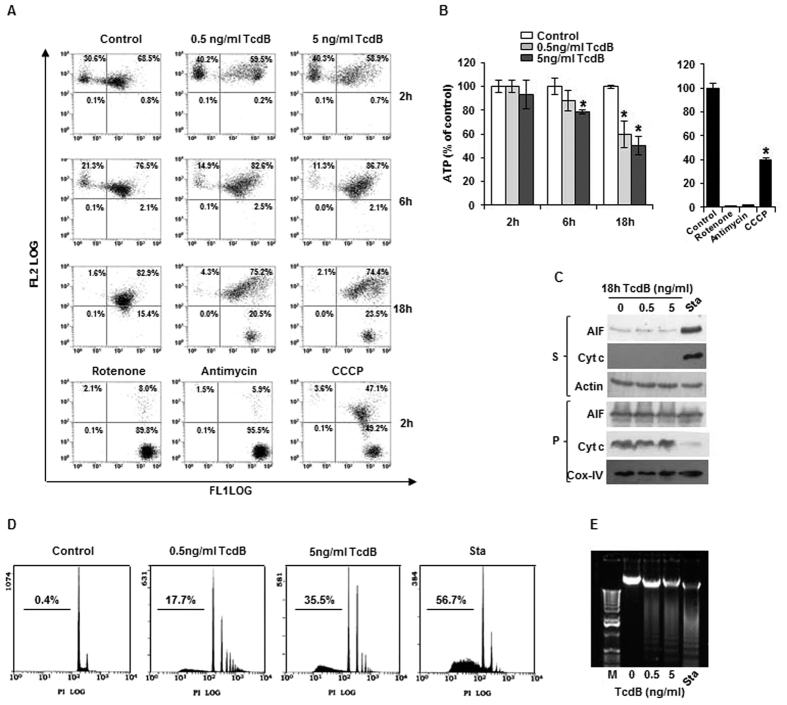
Mitochondrial impairment in TcdB-treated EGCs. (**A**) Cells were treated with TcdB for the indicated times and ∆ψ_m_ was examined by flow cytometric analysis using JC-1. Treatment for 2 hours with rotenone (10 μM), antimycin (30 μM), and CCCP (10 μM) was performed as positive control for ∆ψ_m_ collapse. Each dot represents a single cell analyzed for its green- (FL1) and orange- (FL2) associated fluorescence. (**B**) ATP levels. Data are the mean ± SD of three independent experiments (*p < 0.01 versus control). (**C**) Influence of TcdB on the release of pro-apoptotic factors from mitochondria. TcdB-treated cells were permeabilized with digitonin as described in Materials and Methods and centrifuged (8000 *g* for 10 min). Western blotting of cyt c and AIF was performed in the pellet (P, mitochondrial fraction) and in the supernatant (S, cytosolic fraction). Sta, staurosporine (1 μM), positive control. (**D** and **E**) DNA fragmentation analysis by flow cytometry (**D**) and gel electrophoresis (**E**) confirm cyt c independent apoptosis in EGCs treated for 18 hours with 5 ng/ml TcdB. Sta, staurosporine (1 μM), positive control. In (**A**–**E**) representative experiments out of three are shown.

Late mitochondrial ROS production prompted us to investigate whether TcdB affects mitochondrial energetics. First, we evaluated mitochondrial membrane potential (Δψ_m_) after incubation with 0.5 and 5 ng/ml TcdB for 2–18 hours. Although long lasting incubation (18 hours) produced in control a low decrease of Δψ_m_, which involves homogeneously the total cell pool, in TcdB-treated EGCs a well distinguished cell population (about 20%) shifted to very low Δψ_m_ values (C4 quarter) at 18-hour incubation (Fig. 3A). Treatment for 24 hours did not impair further Δψ_m_ (data not shown). Respiratory chain inhibitors, used as positive controls, produced complete Δψ_m_ collapse. Accordingly, ATP decreased at 18 hours of TcdB treatment and was strongly reduced by the respiratory chain inhibitors (Fig. 3B). It is worth to note that, in the conditions that caused Δψ_m_ decrease, no cytochrome c (cyt c) and apoptosis inducing factor (AIF) release outside mitochondria occurred (Fig. 3C). However, in the same experimental conditions, TcdB induced DNA fragmentation (Fig. 3D,E), demonstrating a cyt c independent apoptotic cell death.

### TcdB regulates the activation of MAPKs and the expression of pro-apoptotic proteins

**Figure 4:**
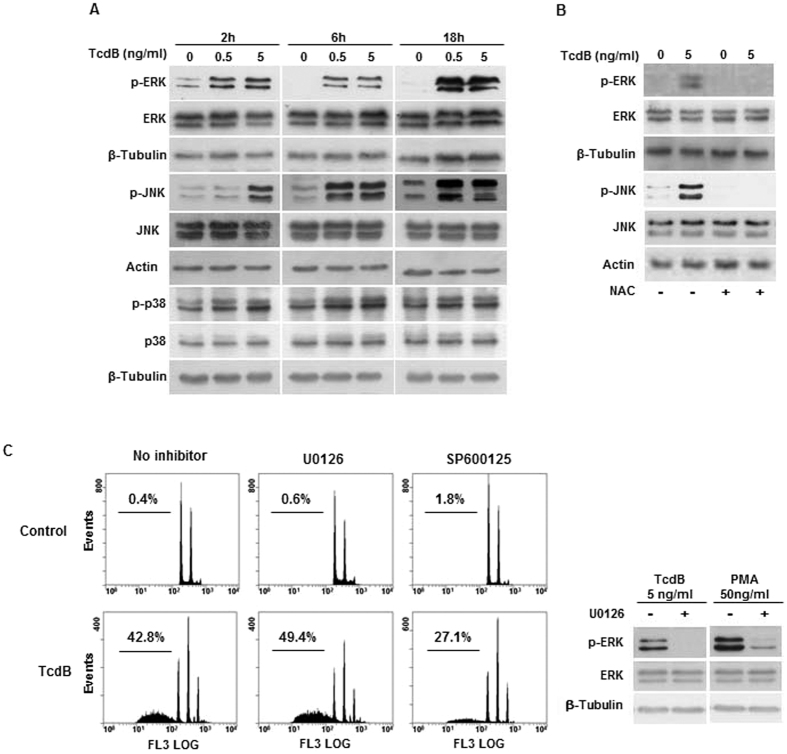
TcdB treatment regulates MAPK activation. (**A**,**B**) EGCs were incubated in the presence of TcdB for the indicated times and protein expression was evaluated by Western blot. In (**B**) NAC (1 mM) was added to cells 1 hour prior to treatment with TcdB for 2 hours. Representative blots of three independent experiments are shown. (**C**) Effect of MAPK inhibitors (10 μM each) on EGCs apoptosis induced by 18-hour treatment with 5 ng/ml TcdB. The Western blot in the right panel shows that U0126 is effective as inhibitor of TcdB or PMA-induced ERK phosphorylation (6-hour treatment). Representative experiment out of three are shown.

ROS have an important role as signal messengers in regulating cellular functions through the activation of MAPKs^19^. MAPK activation was analyzed by Western Blotting, using antibodies for both phosphorylated and total MAPK proteins. Phosphorylation of extracellular signal-regulated kinases (ERK1/2) and c-Jun N-terminal kinase (JNK) occurred at 2-hour treatment with TcdB and persisted until 18 hours (Fig. 4A), without any change in total protein expression. p38 was constitutively phosphorylated and modestly activated by TcdB. As expected, the pretreatment of cells with 1 mM NAC blocked ERK and JNK phosphorylation (Fig. 4B), confirming the role of ROS in MAPK activation.

**Figure 5:**
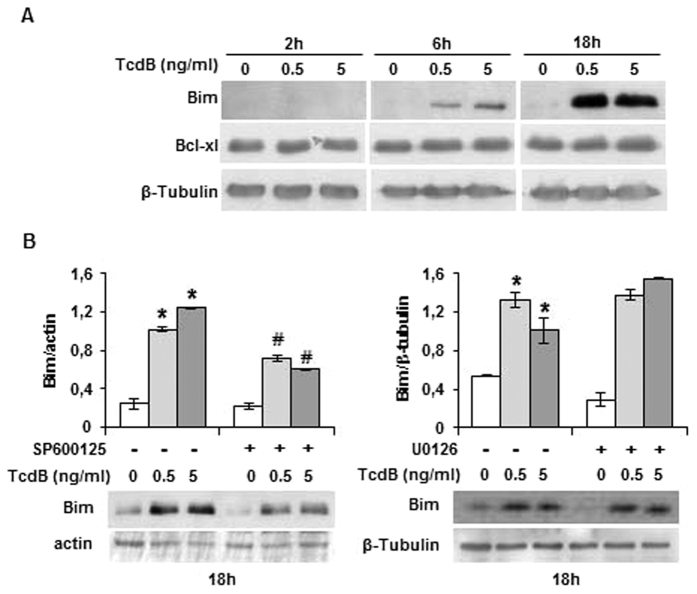
TcdB treatment induces Bim expression. (**A**) EGCs were incubated in the presence of TcdB for the indicated times and protein expression was evaluated by Western blot. (**B**) Effect of MEK/ERK inhibitor (U0126, 10 μM) and JNK inhibitor (SP600125, 10 μM) on Bim expression. Protein expression was analyzed by densitometry. Data are the mean ± SD of three independent experiments (*p < 0.01 versus control; **♯**p < 0.01 versus not inhibited). Representative blots are shown.

To clarify the mechanism of TcdB-induced apoptotic cell death, we studied the effect of selective MAPK inhibitors. Pretreatment with the JNK inhibitor SP600125 reduced the apoptotic cell death, while the MEK/ERK inhibitor U0126 did not exert any protective effect (Fig. 4C), suggesting that TcdB acts through the JNK pathway. JNK interacts with different pro- and anti-apoptotic proteins^20^ of Bcl-2 family members. We reported previously that TcdB did not induce changes in the expression/activation of Bax, Bak, Bcl-2, and Bcl-X_L_^17^. One potential target of JNK is Bim. In neurons undergoing JNK-dependent apoptosis Bim was found transcriptionally up-regulated^21^. In EGCs, Bim increased already at 6-hour TcdB treatment and was elevated at 18 hours, whereas, as already observed, Bcl-X_L_ was not affected (Fig. 5A). The specific JNK inhibitor SP600125 significantly attenuated TcdB-induced Bim expression, whereas the MEK/ERK inhibitor U0126 had no effect (Fig. 5B), indicating that Bim up-regulation occurs through the ROS/JNK signalling pathway.

### ROS-dependent caspases activation

**Figure 6:**
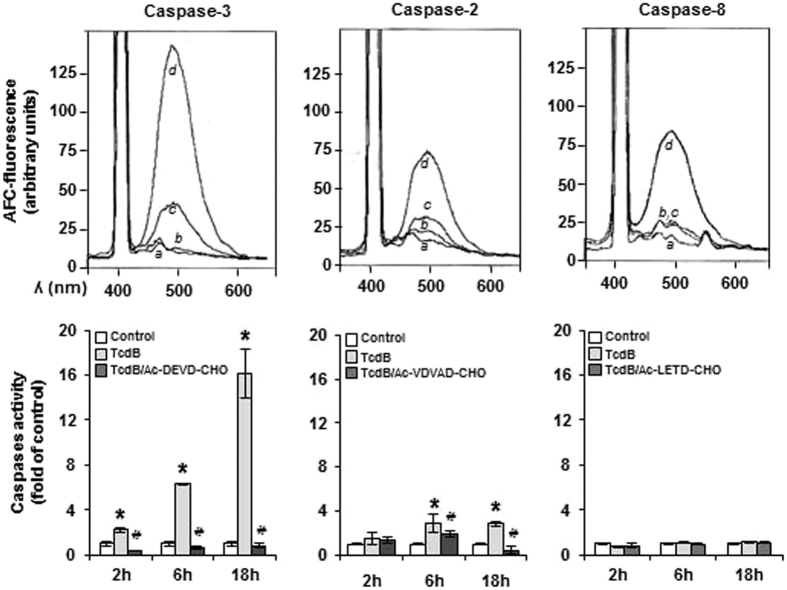
Caspase activity in TcdB-treated EGCs. The activity of caspases was determined in EGCs after 2–18-hour treatment with 5 ng/ml TcdB. Upper panels show representative traces of released AFC fluorescence (λ_ex_ 400 nm) from the specific fluorogenic caspase substrates at 18-hour treatment: (**a**) probe, (**b**) control, (**c**) TcdB, and (**d**) 1 μM staurosporine as positive control. Lower panels show the time course of caspase activity and the effect of the specific -CHO caspase inhibitors that were added to cells 1 h prior to the addition of TcdB. Caspase activity is expressed as fold relative to untreated control cells. Data are the mean ± SD of three independent experiments (*p < 0.01 versus control and ^#^p < 0.01 versus TcdB treatment).

**Figure 7:**
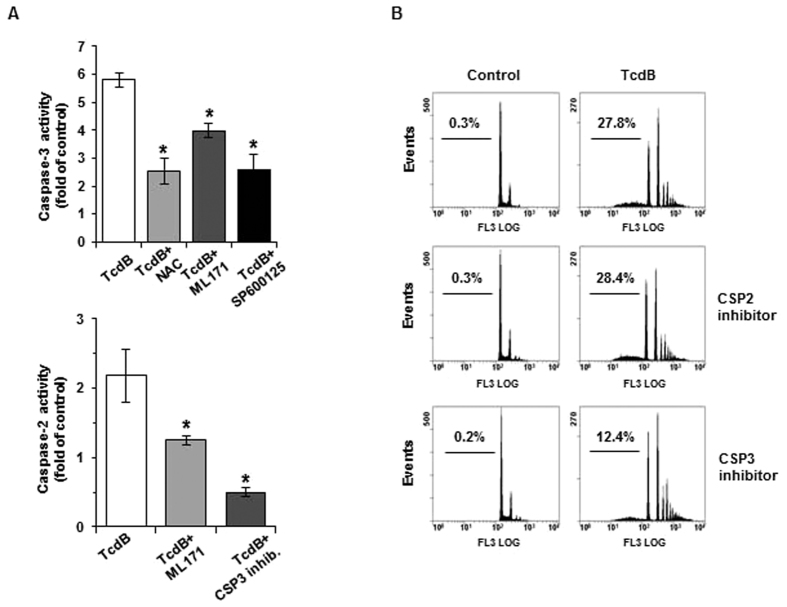
Inhibition of TcdB-induced caspase activity and apoptotic cell death. Cells were treated with 5 ng/mlTcdB in the presence or in the absence of NAC or selected inhibitors. (**A**) Caspase activity at 6-hour treatment, expressed as fold relative to untreated control cells. Data are the mean ± SD of three independent experiments. (**B**) Flow cytometry analysis of PI stained cells at 18-hour treatment. Numbers refer to the percentage of apoptotic cells in each sample. A representative experiment is shown.

Caspase-3 is the early executor of TcdB-induced EGCs apoptosis^17^, which occurs in the absence of cyt c release (Fig. 3C). In view of the central role of caspase-3 and caspase-2 in the apoptotic cascade involving upstream ROS, we investigated whether the ROS/JNK axis could act as a signal for caspases activation in EGCs. Caspase activity was evaluated after treatment with TcdB for 2–18 hours, by monitoring AFC fluorescence that follows the cleavage of the fluorogenic peptides Ac-DEVD-AFC and Ac-VDAVD-AFC, substrates of caspase-3 and caspase-2, respectively. As expected, caspase-3 activity was detectable already at 2 hours and increased noticeably at 6 and 18 hours in treated cells respect to control, whereas caspase-2 followed a different time course, being modestly activated at 6 and 18 hours (Fig. 6). Both caspases were completely inhibited by their specific inhibitors. The possible involvement of an extrinsic pathway in caspase-3 activation was excluded by the lack of caspase-8 activation (Fig. 6). TcdB-induced caspase-3 activity was significantly decreased by the NOX inhibitor ML171 and by the JNK inhibitor SP600125, as well as by the ROS scavenger NAC (Fig. 7A), indicating that caspase-3 activation is triggered by NOX through a pathway involving JNK. At the same time, ML171 and, to a much higher extent the specific caspase-3 inhibitor, reduced capase-2 activation (Fig. 7A), suggesting its dependence on caspase-3. Interestingly, whereas the caspase-3 inhibitor reduced significantly DNA fragmentation, the caspase-2 inhibitor did not exhibit any effect (Fig. 7B), confirming caspase-3 as the executor of apoptotic cell death.

## Discussion

The present study was aimed to define the molecular mechanisms of EGCs defense to TcdB aggressiveness. We found out the involvement of NOX complex, whose activity triggers a downstream apoptotic cascade program, unexpectedly devoid of a significant impact of the toxin on mitochondria, including cyt c release. Therefore, in these cells TcdB evokes a cytoplasmic response.TcdB affects several parameters of EGCs cellular integrity. We found previously that the toxin produces morphological alterations of cytoskeleton and affects cell viability^17^, also demonstrated by the loss of cellular reducing power, as detected by the MTT reduction assay. A peculiar feature of EGCs following TcdB treatment is the early formation of the superoxide O_2_^−^ anion, as well as of total ROS (Figs 1A and 2A). Indeed, a significant level of ROS was detectable at 2 hours and increased at 18 hours, the radical trap NAC favouring their disposal, while the specific NOX inhibitor ML171 stopping their release (Fig. 2C). Since ML171 prevented DNA fragmentation (Fig. 2D), it can be deduced that the apoptotic pathway triggered by the toxin originates from the NOX activity^22^, whereas a minor role can be attributed to the late release of mitochondrial ROS (Fig. 2B). The NOX/ROS pathway activates ERK and JNK, which are components of the serine/threonine MAPK family involved in signal transduction from cell membranes to nucleus^23^. The activation depends on the modification of amino acid residues of protein caused by ROS, although ROS molecular targets have not been clarified^24^. However, it is known that ROS act in thioredoxin oxidation, resulting in the activation of JNK^25^.The p38, a subgroup of MAPK family, is usually activated by several stimuli^26^. In our experimental conditions, p38 is costitutively activated in control EGCs, no apparent effects being exerted by the toxin (Fig. 4A). Therefore, EGCs are in a condition of channelling to apoptotic fate^27^. Of interest, using NAC to reduce free radicals and to block the downstream radical propagation, MAPKs were preserved in dephosphorylated form (Fig. 4B). Once the signal of propagation has reached the two branches of the MAPK family, i.e. ERK and JNK, only the JNK pathway continues, as demonstrated by the fact that the specific JNK inhibitor SP600125, but not the MEK/ERK inhibitor U0126, could significantly attenuate TcdB-induced DNA fragmentation (Fig. 4C). These results are in line with the distinguished role of activated ERK and JNK, the former in regulating cell differentiation and growth, the latter oriented towards stress-responsive gene expression and apoptosis^27^,^28^,^29^.

Components of the Bcl-2 family of proteins are critical death regulators residing upstream of mitochondria^30^. Phosphorylated JNK activates proapoptotic factors of the Bcl-2 family, thus favouring the release of cyt c outside mitochondria, and the downstream cascade that leads to DNA fragmentation^20^. Interestingly, contrarily to what expected^31^, JNK-activated up-regulation of Bim expression in TcdB-treated EGCs (Fig. 5B) does not addresses cells to a mitochondrial apoptotic process. Indeed, the expression of the anti-apoptotic Bcl-X_L_ was invariable, and neither cyt c nor AIF were released (Fig. 3C). Mitochondria of EGCs support almost completely the ATP need, as deduced by the complete ATP collapse that follows treatment with respiratory chain inhibitors (Fig. 3B). In addition, considering that the toxin effect on mitochondrial functionality is restricted to a limited EGCs pool and ATP levels remain sustained, it can be deduced that toxin aggressiveness is mainly confined to the cytosolic compartment, being mitochondria preserved or, at most, involved in the late phase of TcdB treatment.

TcdB does not activate the apical caspase-8 in EGCs, thus excluding an extrinsic apoptotic executory program, and bringing into play other intracellular mechanisms. Pathogenic bacteria can induce cell death through caspase-2^32^,^33^. However, our results ruled out the direct caspase-2 intervention on TcdB-induced apoptosis, based on the observation that caspase-2 activation follows caspase-3 activation (Fig. 6), and the caspase-2 inhibitor is not able to prevent DNA fragmentation (Fig. 7B). Hence, caspase-2 activation seems to be dependent on caspase-3, which is the major player in this scenario^17^. Indeed, caspase-3 activity is detectable as early as at 2 hours and increases over time (Fig. 6), and its inhibition slows DNA fragmentation (Fig. 7B). In addition, the radical trap NAC and inhibitors along the NOX/JNK axis demonstrate the direct involvement of caspase-3 in the process (Fig. 7A), whereas caspase-2 inhibition by the caspase-3 inhibitor indicates the dependence of caspase-2 on caspase-3 (Fig. 7A).

The behaviour of EGCs to contrast enteric bacterial insult should be coerent with the proposed protective role of EGCs. We found that EGCs surviving to TcdB persistently secreted GDNF^17^, which has been demonstrated to play an important role in maintaining the integrity of the intestinal epithelial barrier in normal and pathological conditions^34^. We speculate that the self-protection role of EGCs could also be exerted by activating cytosolic systems as a *buffer* that contrasts the toxin effect, preserving the mitochondrial power plant and sacrificing to death a subpopulation of EGCs. This represent a peculiar EGCs behavior, since in other cellular types, such as CHO cells and human colonocyte, *C. difficile* toxins produce an early extensive mitochondrial dysfunction, resulting in the generation of oxygen radicals and ATP depletion^13^,^14^, whereas in epithelial cells TcdB produces early hyperpolarization of mitochondria^15^. Here we demonstrate that EGCs adopt a cellular defense pathway, typically found in phagocytic cells, in which NOX is activated, producing a respiratory burst.

**Figure 8:**
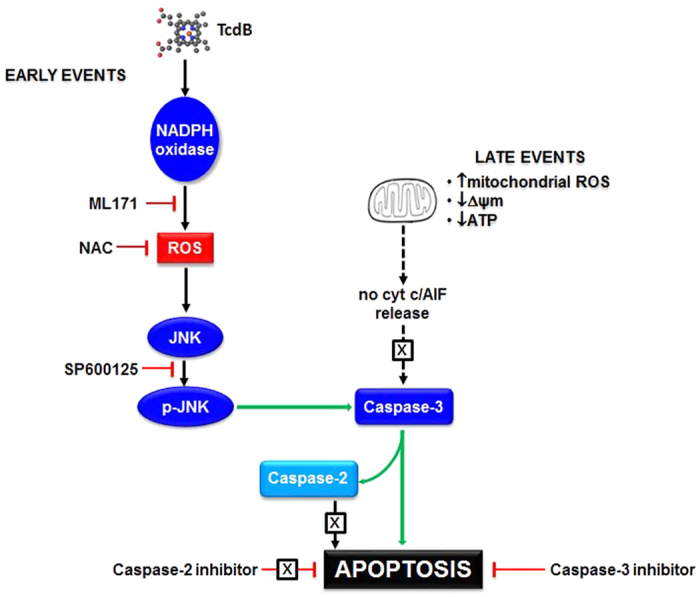
Schematic representation of the pathway activated in EGCs by TcdB.

In conclusion, all our experimental data support the evidence that caspase-3 activity in TcdB-treated EGCs depends on the upstream ROS, through a pathway initiated by NOX and mediated by JNK. These results are in agreement with previous data indicating that caspase-3 may be promoted by ROS, regardless of their mitochondrial or cytoplasmic origin^35^,^36^. Although points remain to be elucidated, such as the lack of mitochondrial apoptosis, despite Bim overexpression, a schematic pathway outlining the cascade of events triggered by TcdB in EGCs is proposed in Fig. 8, which evidences caspase-3 as the promoter of DNA degradation, with NOX/ROS as the initiators throughout the activation of JNK.

## Material and Methods

### Reagents and cells

Rat-transformed EGCs (EGC/PK060399egfr; ATCC^®^ CRL-2690^™^) were obtained from the ATCC (Manassas, VA, USA). Dulbecco’s modified minimum essential medium (DMEM), fetal bovine serum (FBS), penicillin, and streptomycin, were from GIBCO Invitrogen. *C. difficile* toxin B (TcdB) was from Enzo Life Sciences. 5,5′,6,6′-tetrachloro-1,1′,3,3′-tetraethylbenzimidazolylcarbocyanine iodide (JC-1), dihydroethidium (DHE), 5-(and 6)-chloromethyl-2′,7′-dichlorohydrofluorescein diacetate (CM-H_2_DCFDA), and MitoSox Red were from Molecular Probes (Invitrogen, Italy). 3-(4,5-dimethyl-2-thiazolyl)-2,5-diphenyl-2H-tetrazolium bromide (MTT), *N*-Acetyl-L-cysteine (NAC), staurosporine, ML171, Ac-DEVD-AFC, Ac-DEVD-CHO, Ac-VDVAD-AFC, Ac-VDVAD-CHO, Ac-LETD-AFC, Ac-LETD-CHO, phorbol 12-myristate 13-acetate (PMA), and ATP Bioluminescent Assay Kit were obtained from Sigma-Aldrich. U0126 and SP600125 were from Cell Signaling Technology. Antibodies: actin and *β*-tubulin mouse monoclonal (Sigma-Aldrich); cyt c mouse monoclonal, COX-IV mouse monoclonal, AIF goat polyclonal, goat anti-mouse and mouse anti-goat HRP-conjugated IgGs (Santa Cruz Biotechnology); JNK rabbit monoclonal, phospho-JNK (p-JNK) rabbit monoclonal, p44/42 MAPK (ERK1/2) rabbit monoclonal, phospho-p44/42 MAPK (p-ERK1/2) (Thr202/Tyr204) rabbit monoclonal, Bim rabbit polyclonal, Bcl-X_L_ rabbit monoclonal, p38 MAPK rabbit polyclonal, and phospho-p38 MAPK (Thr180/Tyr182) rabbit monoclonal (Cell Signaling Technology).

### Cell culture and treatments

EGCs were grown in DMEM supplemented with 10% heat inactivated fetal bovine serum (FBS), 100 μg/ml streptomycin and 100 U/ml penicillin. Cells were treated with 0.5 or 5 ng/ml TcdB for the indicated times. Staurosporine (1 μM) was a positive control for apoptosis. Where indicated, 1 h prior to the addition of TcdB the following inhibitors were added to cells: 20 μM Ac-DEVD-CHO (caspase-3), 20 μM Ac-VDVAD-CHO (caspase-2), 20 μM Ac-LETD-CHO (caspase-8), 10 μM U0126 (MEK), 10 μM SP600125 (JNK), and 10 μM ML171 (NOX). We verified that these concentrations of each inhibitor did not exhibit any cytotoxic effect in EGCs (data not shown). NAC (1 mM) was added 1 h prior to TcdB as free radical scavenger. In each experiment, controls received treatments, excluding TcdB.

### MTT assay

EGCs (5 × 10^3^/well) were seeded in 96-well plates and treated with TcdB for different times, then MTT was added at 0.5 mg/ml and incubation continued for 2 hours at 37 °C. After removal of the medium, 200 μl DMSO was added to each well. Plates were then shaken for 30 min at 37 °C and the absorbance at 550 nm of reduced MTT was measured using an ELISA reader.

### LDH assay

EGCs (0.5 × 10^6^/well) were seeded in 6-well plates. Released and latent LDH activity were determined in the culture medium and in the cell pellet solubilized with 1% Triton X-100. The reaction rate was determined following NADH oxidation at 340 nm.

### Fluorescent determination of ROS

Cells were treated with TcdB in serum- and phenol red-free DMEM, and then stained for 30 min at 37 °C with fluorescent probes, prior to harvesting. Cellular superoxide ion was determined by flow cytometry (λ_exc_ 488 nm, λ_em_ 630 nm) as fluorescence intensity of oxidized DHE (5 μM). Total intracellular ROS were determined by CM-H_2_DCFDA (10 μM) (λ_exc_ 485 nm, λ_em_ 535 nm). For the detection of mitochondrial superoxide ion, the mitochondrial sensitive dye MitoSox Red (5 μM) was used (λ_exc_ 510 nm, λ_em_ 600 nm).

### Apoptotic cell detection

Apoptotic cells were quantified by flow cytometry analysis of fragmented DNA after PI staining (1 μg/ml) in hypotonic solution, using an EPICS XL-MCL flow cytometer (Beckman Coulter, Miami, FL). Data were processed by an Intercomp and analyzed with Expo 32 software Beckman. For each sample, 10,000 events were recorded and cells with a hypoploid DNA content were quantified as apoptotic cells.

### DNA fragmentation analysis

EGCs (0.5 × 10^6^/well) were seeded in 6-well plates and treated with TcdB. After harvesting, the cells were incubated in 250 μl digestion buffer (10 mM Tris–HCl, pH 7.4, 10 mM EDTA, 100 mM NaCl, 0.5% SDS, and 250 μg/ml proteinase K) at 50 °C overnight, followed by the addition of RNAase A (250 μg/ml). DNA was extracted by phenol–chloroform–isoamyl alcohol (25:24:1), subjected to electrophoresis on a 0.8% agarose gel at 70 V for 1 h, and visualized by ethidium bromide staining.

### Cytofluorimetric analysis of mitochondrial membrane potential

Δψ_m_ was determined by using the JC-1 fluorescent probe (7.5 μM), which selectively enters into mitochondria and changes color from green to orange following Δψ_m_ increase. JC-1 fluorescence of mitochondria was detected as described^37^, and red/green fluorescence emission (FL1/FL2) of particles was reported as a dot plot. The respiratory chain inhibitors rotenone (10 μM), antimycin (30 μM), and the uncoupler CCCP (10 μM) were used to provide complete depletion of Δψ_m_ (positive control). Flow cytometry analysis was performed using an EPICS XL-MCL (Beckman Coulter). Data were analyzed by a data management system (Expo 32 software, Beckman Coulter, UK). Green (FL1) and red (FL2) fluorescence emission of particles are reported.

### ATP determination

ATP levels were quantified by the ATP Bioluminescent Assay (FL-AA, Sigma) by using a calibrated ATP standard curve.

### Cytochrome c release

EGCs (5 × 10^6^) were resuspended in PBS and permeabilized with digitonin (0.1 mg/mg protein). After incubation for 10 min at 0 °C, post-digitonin supernatants were recovered by centrifuging (8,000 *g*, 10 min), whereas pellets were resuspended in a proper amount of PBS. Western blot analysis of cyt c and AIF was performed in pellet and supernatant. The efficiency of permeabilization was evaluated by PI staining of DNA.

### Western blotting

Cell lysates were prepared using lysis buffer (1% SDS, 1 mM Na-vanadate, 10 mM Tris-HCl pH 7.4) in the presence of 0.1 mM phenylmethylsulfonyl fluoride and protease inhibitor cocktail. Proteins (40 μg) were subjected to SDS-PAGE and electroblotting on nitrocellulose membranes, which were probed with specific primary and HRP-conjugated secondary antibodies. Immunoblots were revealed by enhanced chemiluminescence reagent (Bio-Rad). Images were acquired using the VersaDoc 1000 imaging system and individual band densities were integrated by Quantity One software (BioRad).

### Caspase activity

Caspase activity was assayed as previously described^37^. Cell suspensions (2 × 10^6^, about 300 μg protein in 100 μl) were lysed with 100 μL of lysis buffer containing 50 mM HEPES (pH 7.4), 10% sucrose, 0.1% Triton X-100, 5 mM EDTA, and 5 mM EGTA and incubated 40 min in ice. After centrifugation for 10 min at 9,000 *g* pellet was discarded and supernatant incubated 30 min at 30 °C in the presence of 2.5 mM DTT. The caspase-specific fluorogenic substrate Ac-DEVD-AFC (caspase-3), Ac-VDVAD-AFC (caspase-2), and Ac-LETD-AFC (caspase-8) was then added (20 μM) and incubation continued for 30 min. Samples were then added to 1.8 ml of PBS and elicited AFC fluorescence was recorded (λ_exc_ 400 nm and λ_em_ 505 nm), using a Shimazu RF-500 spectrofluorometer equipped with temperature control and magnetic stirrer device.

### Statistical analyses

The results, expressed as means ± SD of at least three independent experiments, were analyzed for statistical significance by Student’s *t*-test. p-values < 0.05 were considered significant.

## Additional Information

**How to cite this article:** Macchioni, L. *et al*. Enteric glial cells counteract *Clostridium difficile* Toxin B through an NADPH oxidase/ROS/JNK/caspase-3 axis, without involving mitochondrial pathways. *Sci. Rep.* **7**, 45569; doi: 10.1038/srep45569 (2017).

**Publisher's note:** Springer Nature remains neutral with regard to jurisdictional claims in published maps and institutional affiliations.

## Supplementary Material

Supplementary Figure S1
