# Supplementary material for: Enteric glial cells counteract Clostridium difficile Toxin B through a NADPH oxidase/ROS/JNK/caspase-3 axis, without involving mitochondrial pathways
Source: Sci Rep. 2017 Mar 28;7:45569. doi: 10.1038/srep45569 (PMC5368562; doi:10.1038/srep45569)
Supplement: Supplementary Figure S1 [file srep45569-s1.pdf]

Enteric glial cells counteract *Clostridium difficile* Toxin B through an NADPH oxidase/ROS/JNK/caspase-3 axis, without involving mitochondrial pathways

Lara Macchioni[1], Magdalena Davidescu[2], Maya Petricciuolo[1], Leonardo Gatticchi[2], Katia Fettucciari[1], Davide Gioè[1], Vincenzo Villanacci[3], Massimo Bellini[4], Pierfrancesco Marconi[1], Rita Roberti[1], Gabrio Bassotti[5] & Lanfranco Corazzi[1*]

[1]Department of Experimental Medicine, University of Perugia, Perugia, Italy, [2]Scientific and educational center of Terni, University of Perugia, Perugia, Italy, [3]Pathology Section, Spedali Civili di Brescia, Brescia, Italy, [4]Department of Gastroenterology, University of Pisa, Pisa, Italy, [5]Department of Medicine, University of Perugia, Perugia, Italy.

*Corresponding author: Lanfranco Corazzi, Department of Experimental Medicine, University of Perugia, 06132 Perugia, Italy. Tel.: 39 075 5858196; e-mail: lanfranco.corazzi@unipg.it

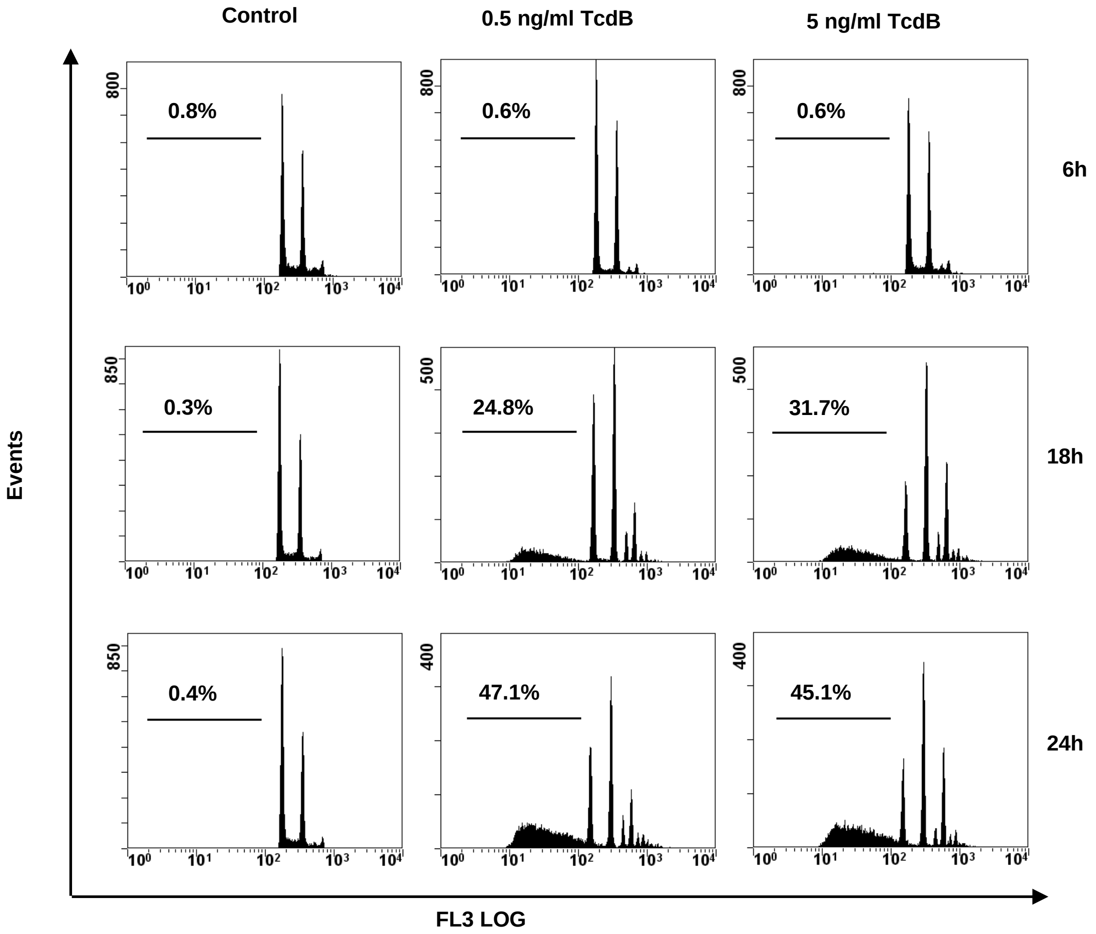

Figure S1. TcdB induces cell death in EGCs. Cells were treated with of 0.5 or 5 ng/ml TcdB for the indicated times. Flow cytometric analysis of EGCs showed that TcdB did not cause the decrease of propidium iodide fluorescence at 6h but was effective at 18 and 24 hour treatment. Data are expressed as percentage of hypodiploid nuclei. A representative experiment out of three is shown.
